# Supplementary material for: Associations between Tobacco Use, Surges, and Vaccination Status over Time in the COVID-19 Era
Source: Int J Environ Res Public Health. 2023 Jan 9;20(2):1153. doi: 10.3390/ijerph20021153 (PMC9859008; doi:10.3390/ijerph20021153)
Supplement: Supplementary file 1 [file ijerph-20-01153-s001.zip › ijerph-2107243-supplementary.pdf]

Table S1. Multinomial model predicting daily and non-daily tobacco use for visits occurring when the vaccine was available.

	Daily tobacco use vs. nonuse				Non-daily tobacco use vs. nonuse			
**Predictor**	**Coeff.**	**SE**	**O.R.**	**95% CI**	**Coeff.**	**SE**	**O.R.**	**95% CI**
Intercept	-2.83	0.15	-	-	-3.51	0.23	-	-
Time	0.02	<0.01	1.02	1.01, 1.02	0.02	<0.01	1.02	1.01, 1.02
Vaccination status	-0.31	0.02	0.73	0.70, 0.76	-0.15	0.03	0.87	0.81, 0.92
Surge	-0.13	0.02	0.88	0.85, 0.91	-0.08	0.03	0.93	0.88, 0.98
Race								
Black/AA	0.18	0.02	1.20	1.15, 1.25	0.50	0.03	1.65	1.55, 1.76
Asian American	-0.51	0.04	0.60	0.56, 0.64	-0.21	0.05	0.81	0.73, 0.90
NH/PI	-0.11	0.06	0.90	0.80, 1.00	0.09	0.09	1.09	0.93, 1.29
AI/AN	0.01	0.07	0.99	0.86, 1.14	0.01	0.11	1.01	0.82, 1.25
Other	-0.20	0.03	0.82	0.77, 0.87	<0.01	0.04	1.00	0.92, 1.09
Ethnicity								
Hispanic	-0.59	0.03	0.56	0.53, 0.58	0.08	0.03	1.09	1.02, 1.16
Sex	-0.54	0.03	0.58	0.55, 0.62	-0.93	0.05	0.39	0.35, 0.44
Age	-0.01	<0.01	0.99	0.99, 0.99	-0.03	<0.01	0.97	0.97, 0.98
Visit Number	0.01	0.02	1.01	0.97, 1.04	-0.11	0.03	0.90	0.85, 0.95
VA location	<0.01	<0.01	1.00	1.00, 1.00	<0.01	<0.01	1.00	1.00, 1.00

Note: The following categories served as reference groups: No history of vaccination, visit not occurring during a COVID-19 surge, being Caucasian, being non-Hispanic, and being male.
